# Supplementary material for: Chromothripsis during telomere crisis is independent of NHEJ, and consistent with a replicative origin
Source: Genome Res. 2019 May;29(5):737–49. doi: 10.1101/gr.240705.118 (PMC6499312; doi:10.1101/gr.240705.118)
Supplement: Supplemental Material [file supp_gr.240705.118_Supplemental_file_1.zip › contigs/annotated_contigs/DB57/contig.2.DB57_length_263_mean_cov_3.99239543726.docx]

**DB57_length_263_mean_cov_3.99239543726**

```
TATTTCTTTTGGCGAAATGAAACACCTGCAGGCCACATCTGGTCCGCAGGTGACCAGTGAGTGGTGTTCACTTGAATCAATATACCAGA
 >chr7:154616763-154616999 + E=2e-131
TGGCCAAAGAGGACTTCTTCCTGTTTCTCAGCCTTGCTTACAAGCTACTCACTTGGTCATTAAGAAATCACCAAAGCTTATGTAGCCAG

AGGGAGCCATAACAGAAGTGGATGTGCTTAAGTGTCAAGGATCCAACCTGGACACT|CA|AAGGACATGAACATACAGTTCTCAAAA                                                         >chr7:156495117-156495146 -
```
